# Current Attitudes toward Unfunded Cancer Therapies among Canadian Medical Oncologists

**DOI:** 10.3390/curroncol28060400

**Published:** 2021-11-16

**Authors:** Selina K. Wong, Lovedeep Gondara, Sharlene Gill

**Affiliations:** 1 Departments of Medical Oncology, British Columbia Cancer Agency, Vancouver, BC V5Z 4E6, Canada; selina.wong1@bccancer.bc.ca; 2 Departments of Cancer Surveillance and Outcomes, British Columbia Cancer Agency, Vancouver, BC V5Z 4E6, Canada; lovedeep.gondara@bccancer.bc.ca

**Keywords:** unfunded cancer therapies, Canadian, medical oncology

## Abstract

Background: Despite successes in the development of innovative anticancer therapies, the fiscal and capacity restraints of the Canadian public healthcare system result in challenges with drug access. A meaningful proportion of systemic therapies ultimately do not receive public funding despite supporting clinical evidence. In this study, we assessed Canadian medical oncologists’ current attitudes toward discussing publicly unfunded cancer treatments with patients and predictors of different practices. Methods: A web-based survey consisting of multiple choice and case-based scenarios was distributed to medical oncologists identified through the Royal College of Physicians and Surgeons of Canada directory. Results: A total of 116 responses were received. Almost all respondents reported discussing publicly unfunded treatments, including those who did so for Health Canada (HC) approved treatments (50%) and those who discussed off-label treatments (i.e., not HC approved) as guided by national guidelines (48%). Respondents in practice for over 15 years versus less than 5 years (OR 0.14, 95% CI 0.04–0.50, *p* = 0.002) and those who worked in a community practice versus comprehensive cancer center (OR 0.17, 95% CI 0.03–0.91, *p* = 0.04) were significantly less likely to discuss off-label treatment options with their patients. Almost half of respondents (47%) indicated that their institution did not permit the administration of unfunded treatments. Conclusions: There is variability in medical oncologists’ practices when it comes to discussing unfunded therapies. Given the limitations within Canada’s publicly funded healthcare system, physicians are faced with the challenge of navigating an increasingly complex balance between patient care and available resources. Engagement of relevant stakeholders and policy makers is crucial in the continued evaluation of Canada’s drug funding process.

## 1. Introduction

The treatment of cancer is becoming increasingly complex with continued discovery of novel agents and incorporation of multimodality therapy. While innovative treatments such as targeted therapy and immunotherapy have revolutionized our approach to many cancers and improved patient outcomes, public healthcare systems such as that of Canada are consequently faced with the challenge of drug funding and access. An analysis of the economic burden of cancer care in Canada done in 2018 found a greater than doubling from $2.9 billion in 2005 to $7.5 billion in 2012 [1]. In Canada, the sequence of events from scientific discovery to public funding begins with Health Canada approval, followed with assessment by the pan-Canadian Oncology Drug Review (pCODR) committee, and subsequently provincial-level funding decisions [2,3]. As a result of fiscal and capacity limitations, a certain proportion of evidence-based treatments will not receive approval for public funding despite demonstrated clinical benefit. Medical oncologists must navigate these discrepancies between international guidelines, regulatory body approval, and publicly funded treatments within their specific province of practice. Our objectives for this study are to describe current attitudes toward and examine predictors of discussion of unfunded cancer treatments among Canadian medical oncologists.

## 2. Materials and Methods

### 2.1. Population

Canadian medical oncologists were identified using the Royal College of Physicians and Surgeons of Canada (RCPSC) directory. RCPSC is a national regulatory body that requires all specialists, including medical oncologists, to be certified in order to obtain a license to practice. After institutional review board approval, an English web-based survey was created on Research Electronic Data Capture (REDCap^©^) and oncologists were invited to participate through email. Respondents were surveyed from 7 to 31 July 2020. Reminders were sent via email. While no direct incentive was offered, an in-kind donation of $15 per completed survey was made to the Canadian Cancer Society. 

### 2.2. Survey

The survey was created by the authors, consisting of multiple-choice questions and three case-based scenarios involving Health Canada approved unfunded therapies (Supplemental Materials). Multiple choice questions were designed to elucidate basic information regarding oncologists’ demographics, practice, and experience with unfunded therapies (e.g., access to a drug access navigator, impact on workload, and level of concern regarding the future of drug funding). The case-based scenarios were designed to offer real-world examples for respondents, with variations between scenarios to capture how changes in factors such as cost of drug or route of administration might impact responses. Given the nature of this survey, our intent was to offer a descriptive analysis of current oncology practices.

### 2.3. Statistical Analysis

Variable distribution was studied with the help of descriptive statistics. To model the association between discussing off-label, unfunded treatment options with practice location (geographical area), practice setting, sex, years in practice, institutional constraints, workload, and availability of a drug access navigator, a multivariable logistic regression model was used, with the alpha level set at 0.05 for statistical significance. All analyses were conducted in R (version 4.0.2) [4]. 

## 3. Results

A total of 116 medical oncologists completed the survey (23% response rate): 53% female, most respondents practiced in British Columbia (35%) or Ontario (27%), 88% practiced at a comprehensive cancer center versus community practice, 47% had been in practice for over 15 years, and 39% had completed some training outside Canada (Table 1). 

**Figure 1:**
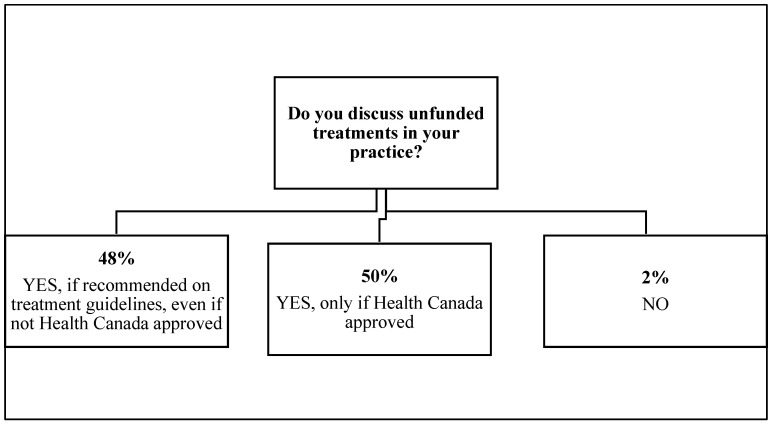
Patterns of practice discussing unfunded treatments among respondents.

In response to the question of “do you discuss provincially unfunded treatments with your patients,” 98% responded in the affirmative with 48% indicating that they discuss unfunded treatments if included in national guidelines even if not approved by Health Canada (i.e., off-label) and 50% indicating only discussing unfunded treatments that are Health Canada approved. Only 2% reported never discussing unfunded treatments in their practice (Figure 1). 

Between the two cohorts who reported discussing unfunded treatments in their practice, but differed in whether or not they discussed off-label treatments, respondents in practice >15 years compared to <5 years were significantly less likely to discuss off-label treatments (odds ratio [OR] 0.14, 95% confidence interval [CI] 0.04–0.50, *p* = 0.002. Similarly, those working in a community cancer center compared to those working in a comprehensive cancer center were significantly less likely to discuss off-label treatments (OR 0.17, 95% CI 0.03–0.91, *p* = 0.04) (Table 2). 

The majority of respondents (63%) felt that discussing unfunded cancer treatments had a moderate to significant impact on their workload (greater than 15 min/patient). Eighty-four percent had access to a drug access navigator at their institution. While approximately half of respondents (49%) estimated that 20–40% of their patients have private health insurance, the majority (62%) reported that less than 10% of their patients are on unfunded treatments. As for institution-specific regulations, 47% indicated that their institution does not permit administration of unfunded treatments, 29% were permitted to administer unfunded treatments at their institution only if accessed through a manufacturer access program, and 23% reported that unfunded treatments are permitted at their institution. Most respondents (90%) expressed moderate to extreme concern regarding the future of drug funding in Canada, compared to 11% who were only slightly or not concerned.

**Figure 2:**
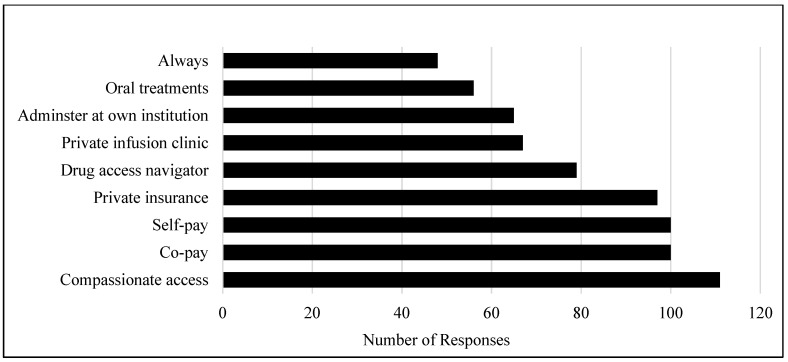
Response to the question “I would prescribe provincially unfunded treatments if…” (select all that apply).

The factors most likely to influence respondents’ decision towards discussing unfunded treatments included availability of a compassionate access program, manufacturer co-pay, when patients express interest in self-pay options, and if patients have private insurance (Figure 2). 

The three case-based scenarios are described in detail in the supplemental material. In the first case of mismatch repair deficient metastatic colorectal cancer, 52% of respondents indicated that they would always discuss pembrolizumab versus 9% who would never discuss this option. The top two reasons as to why respondents chose never to discuss this option were “I am worried it would cause increased stress for the patient and their family” and “I don’t think the patient could afford it.” In the second case of chemotherapy refractory metastatic colorectal cancer, 43% of respondents indicated that they would always discuss the option of trifluridine/tipiracil versus 11% who would never discuss the option due to “I am not convinced that this treatment offers a meaningful benefit” and “I am worried it would cause increased stress for the patient and their family.” Finally, the third case of larotrectinib in NTRK fusion positive cancer, 44% of respondents would always discuss this option versus 9% who would never discuss. The top two reasons against discussing this treatment option were “I am worried it would cause increased stress for the patient and their family” and “I don’t think the patient could afford it.” In all three cases among respondents who indicated that they would consider discussing unfunded treatments only in certain scenarios, patients expressing that they would consider self-pay options was the most common reason to offer unfunded treatments.

## 4. Discussion

In our survey of medical oncologists practicing in Canada, almost all respondents (98%) reported discussing publicly unfunded treatments in their practice. Among these, a subset of almost half reported not only discussing unfunded treatments, but also those included in national guidelines yet not Health Canada approved and thus considered off-label. Respondents in practice for over 15 years compared to less than 5 years and those who worked in a community practice compared to comprehensive cancer center were significantly less likely to discuss off-label treatment options with their patients. We did not find an association between geographic location, gender, workload, or institutional drug access navigator and likelihood of discussing off-label treatments.

The challenges faced by oncologists navigating the discrepancies between scientific advances, guideline recommendations, regulatory approvals, and funding of treatments have been examined previously. In Canada, a study by Chan et al. evaluated the discussion and use of bevacizumab as part of first-line treatment of metastatic colorectal cancer (CRC). Although bevacizumab gained Health Canada approval in 2005, only two provinces in Canada funded bevacizumab at the time of the study in 2007. Less than 30% of oncologists felt they were able to use what they considered to be the ideal first-line regimen for their patients, only 18% could use bevacizumab routinely, and less than 45% reported always discussing the role of adding bevacizumab to their patients [5]. 

Despite being backed by scientific evidence, there are a large number of unfunded treatment regimens. A study based in Australia evaluated 448 protocols across 15 tumor groups finding that 42% of the protocols were off-label and therefore unfunded. Of these unfunded treatments, 91% of them were supported by evidence-based treatment guidelines or published phase II or III clinical trial data [6]. Physicians who try to obtain access to unfunded treatments do so by different means such as private insurance, clinical trials, compassionate access programs and even falsifying claims or using leftover drug supplies [7]. We similarly observed a high reliance on compassionate access and co-pay programs, with these two being the top reasons that would sway a respondent towards discussing unfunded options with their patients. Inevitably, this not only increases time and effort on the part of the oncologist, but can also have a negative impact on the physician-patient relationship [8]. As described by one respondent in our survey, “this type of situation is extremely morally draining for physicians.”

Physicians’ concerns regarding the potential for discussion of unfunded cancer treatments to cause psychological and/or financial harm to patients have been reported in other studies and was similarly described in our survey [9,10]. Sixty-two percent of respondents estimated that less than 10% of their current practice consisted of patients on unfunded therapy. Different factors are weighed when considering whether or not to discuss unfunded options, including patient autonomy, level of evidence, specific patient population, and the risk of causing harm. Below are some comments made by respondents:

“I might bring it up even if I was not sure if they could pay, sometimes it’s hard to tell what financial resources people have…”

“Phase 1/2 trial data not sufficient…”

“I feel that it is important to give the patient all the appropriate available treatment options…but the degree to which I discuss a non-funded treatment can vary.”

“I would try to present information in a manner that wouldn’t be a torment to the patient, like dangling something unobtainable that might be life-saving”

“I am very concerned with burdening patients with a possible treatment that they cannot afford/access. Has potential to cause harm.”

Although the development of pCODR was a step towards improved uniformity across Canada, the final decision with drug funding still lies with each province. As a result, there is inter-provincial variability with respect to the funding of therapies and accessing unfunded therapies. In a Canadian survey, Alberta was found to have significantly higher rates of access to unfunded treatments in respondents’ own institutions compared to Ontario, but lower rates of access in private clinics. In contrast, British Columbia had lower rates of access to unfunded drugs at their own institutions compared to Ontario [11]. Even after drugs are approved for public funding, variability between provinces in terms of accessing these cancer drugs have been described [12]. Our study did not evaluate interprovincial variability regarding access to unfunded drugs, but no significant difference was found in patterns of practice discussing off-label, unfunded treatments between British Columbia and Ontario, which were our two largest groups of respondents. 

Patient perspectives and preferences play a major role in physician decision-making. In our study, patients expressing a desire to know about self-pay options was one of the top three reasons why respondents would choose to discuss unfunded cancer treatments. Likewise in our case-based scenarios, among respondents who reported that they would not routinely discuss the unfunded treatments with their patients, the top reason across all three cases as to why they would be motivated to do is if the patient expressed interest in self-pay options. In an evaluation of patients’ and the general public’s perspective on the issue of unfunded anticancer therapies, a study in the United Kingdom found that the majority of both groups, 94% of patients and 93% of the general public, felt that doctors should tell patients about all available cancer treatments even if not publicly funded [13]. Mileshkin et al. found similarly that the Australian general public want to be informed of unfunded, expensive anti-cancer drugs regardless of willingness or ability to pay for them [14].

Not surprisingly, medical oncologists are concerned regarding the future of drug funding in Canada with over half of the respondents in our survey expressing “extreme” concern. In addition to remaining up-to-date on scientific advances, novel therapies, and continually changing treatment recommendations, oncologists are simultaneously faced with the challenge that many evidence-based treatments do not gain approval for public funding. Even for those that are eventually approved, funding often lags behind scientific discovery. While some oncologists argue that the discussion of treatment costs should not fall under physician responsibilities during clinical encounters and others reporting discomfort having to discuss costs with patients [15], a shift in responsibilities may become inevitable to align with the expectations of patients and the general public. Accordingly, physicians will require the necessary knowledge concerning costs of care, tools, and resources [16]. This should include education around communicating funding discussions with patients, as well as recognition of the increased workload on medical oncologists associated with the growing proportion of patients requiring navigation to access unfunded therapies.

Our findings offer a current examination of Canadian medical oncologists’ attitudes toward the discussion of unfunded and off-label anticancer treatments. It offers a unique, contemporary perspective of current oncology practices, demonstrating variability, highlighting challenges faced by clinicians, and offering data from which more rigorous, prospective studies may be undertaken to inform Canada’s drug funding policies. There are also limitations to our study; firstly, recall bias is a limitation wherein respondents are asked to describe their past and current practices. Secondly, the findings from our survey may not be representative of all Canadian medical oncology practices due to non-response bias and the majority of respondents being in practice at a comprehensive cancer center. We also acknowledge that due to the nature of this survey-based observational study, the discrepancy between the number of respondents from comprehensive cancer centers versus community practices (102 vs. 14) may impact analyses. 

## 5. Conclusions

Given fiscal and capacity limitations within Canada’s publicly funded healthcare system, coupled with the increasing prevalence of complex cancer cases, it is anticipated that a growing proportion of cancer therapies may not receive approval for public funding in Canada. Our survey reveals variability in contemporary practice with respect to medical oncologists’ patterns of practice when it comes to discussing unfunded therapies, with greater years in practice and working in a community practice being negative determinants of likelihood to discuss off-label, unfunded treatment options. Engagement of relevant stakeholders and continued evaluation of Canada’s drug funding process are necessary as physicians will face increasing difficulty bridging the gap between the available resources within the healthcare system and the needs as well as preferences of individual patients with cancer.

## Figures and Tables

**Table 1:** Respondent demographics.

Demographic	N (%)
Gender	
Female	62 (53)
Male	53 (46)
Not disclosed	1 (1)
Province of practice	
British Columbia	41 (35)
Ontario	31 (27)
Alberta	13 (11)
Quebec	12 (10)
Manitoba	6 (5)
New Brunswick	6 (5)
Nova Scotia	3 (3)
Saskatchewan	2 (2)
Newfoundland	1 (1)
Prince Edward Island	1 (1)
Practice setting	
Comprehensive Cancer Center	102 (88)
Community	14 (12)
Private Practice	0 (0)
Disease site	
Gastrointestinal	55 (47)
Breast	55 (47)
Genitourinary	34 (29)
Lung	34 (29)
Gynecologic	22 (19)
Melanoma	22 (19)
Sarcoma	18 (16)
Head and Neck	19 (16)
Hematology/Lymphoma	18 (16)
Other	20 (17)
Years in practice	
<5 years	22 (19)
5–10 years	27 (23)
10–15 years	13 (11)
>15 years	54 (47)
Previous training outside Canada	
Yes	45 (39)
No	71 (61)

**Table 2:** Likelihood of discussing off-label, unfunded treatment options.

Variable	Odds Ratio (95% Confidence Interval)	*p*-Value
Ontario vs. British Columbia	1.53 (0.47–4.97)	0.47
Quebec vs. British Columbia	0.70 (0.12–3.91)	0.68
Atlantic vs. British Columbia	0.99 (0.16–6.19)	0.99
Prairies vs. British Columbia	0.20 (0.04–0.87)	0.03 *
Community vs. Comprehensive Cancer Center	0.17 (0.03–0.91)	0.04 *
Male vs. Female	2.35 (0.92–5.97)	0.07
Years in practice 5–10 y vs. <5 y	0.40 (0.10–1.58)	0.19
Years in practice 10–15 y vs. <5 y	0.24 (0.04–1.27)	0.09
Years in practice >15 y vs. <5 y	0.14 (0.04–0.50)	0.002 *
Institution permits administration (Only if manufacturer access program vs. Yes	0.43 (0.12–1.59)	0.20
Institution permits unfunded treatment, No vs. Yes	0.49 (0.14–1.74)	0.27
Workload (No/Minimal vs Moderate/Significant ^#^)	0.42 (0.15–1.16)	0.09
Drug access navigator available, No vs. Yes	2.29 (0.52–10.15)	0.28

* reached statistical significance. ^#^ Minimal impact on workload defined as <15 min per patient, moderate 15–30 min per patient, and significant >30 min per patient.

## Data Availability

The data presented in this study are available on request from the corresponding author. The data are not publicly available due to survey participants’ privacy.

## Supplementary Materials

The following are available online at https://www.mdpi.com/article/10.3390/curroncol28060400/s1, Description of the three case-based scenarios included in the survey.
