# Supplementary material for: Current Attitudes toward Unfunded Cancer Therapies among Canadian Medical Oncologists
Source: Curr Oncol. 2021 Nov 16;28(6):4748–55. doi: 10.3390/curroncol28060400 (PMC8628663; doi:10.3390/curroncol28060400)
Supplement: Supplementary file 1 [file curroncol-28-00400-s001.zip › curroncol-1415008-supplementary.pdf]

Given increasing demands in the face of escalating fiscal and capacity restraints in the Canadian healthcare system, it is recognized that a proportion of anti-cancer systemic therapies may not be publicly funded despite supporting clinical evidence and, in many cases, Health Canada regulatory approval. This challenge of unfunded cancer therapies has grown in the era of emerging novel therapies, tumour-agnostic approaches, broader genomic testing and immunotherapies.

*** For the purposes of this survey, “provincially unfunded therapies” is defined as a therapy which is not publicly-funded (including therapies that are available through a manufacturer compassionate access programs) ***

In which province do you practice?

- ◯ British Columbia
- ◯ Alberta
- ◯ Saskatchewan
- ◯ Manitoba
- ◯ Ontario
- ◯ Quebec
- ◯ Newfoundland
- ◯ New Brunswick
- ◯ Nova Scotia
- ◯ Prince Edward Island

Please describe your practice setting:

- ◯ Comprehensive cancer center
- ◯ Community hospital-based practice
- ◯ Private practice
- ◯ Other

What is your practice setting?

____

Which of the following disease sites do you treat? (Select all that apply)

- ☐ Gastrointestinal
- ☐ Genitourinary
- ☐ Lung
- ☐ Breast
- ☐ Gynecology
- ☐ Sarcoma
- ☐ Head and neck
- ☐ Melanoma
- ☐ Hematology/Lymphoma
- ☐ Other

What is your sex/gender?

- ◯ Female
- ◯ Male
- ◯ Prefer not to disclose

How many years have you been in practice as a medical oncologist?

- ◯ Less than 5 years
- ◯ 5-10 years
- ◯ 10-15 years
- ◯ More than 15 years

Please estimate the proportion of your patients who have extended health insurance?

- ◯ Less than 20%
- ◯ 20-40%
- ◯ 40-60%%
- ◯ greater than 60%

Have you previously trained or practiced outside of Canada?

- ◯ Yes
- ◯ No

In your current practice, do you discuss provincially unfunded treatments with your patients?	◯ Yes -- if recommended by a national guideline (e.g. ASCO, NCCN, ESMO), even if NOT Health Canada approved<br>◯ Yes -- if it is Health Canada approved<br>◯ No -- I only discuss provincially funded treatment options
What is your reason for not discussing provincially unfunded treatments? (Select all that apply)	☐ Patient perception (i.e. patient frustration discussing a treatment that is not funded)<br>☐ It increases my workload (i.e. additional appointment time, paperwork, etc)<br>☐ Limited resources/capacity to provide treatment (e.g. unit bed space)<br>☐ Unfunded treatments do not offer a clinically meaningful benefit worth offering<br>☐ Other (please describe below)
Please describe:	__________________________________
How does discussing unfunded treatments affect your clinical workload?	◯ No effect on my work load<br>◯ It increases my work load minimally (< 15 mins/patient)<br>◯ It increases my work load moderately (15-30 mins/patient)<br>◯ It increases my work load significantly (>30 mins/patient)
I would prescribe provincially unfunded treatments if: (Select all that apply)	☐ There is a drug access navigator at my center<br>☐ There is a manufacturer compassionate access program<br>☐ There is a manufacturer access program with co-pay coverage<br>☐ It is an oral therapy<br>☐ It is an IV therapy and I have access to a private infusion clinic<br>☐ It is an IV therapy and I can administer in my own institution<br>☐ The patient has private insurance<br>☐ The patient is very motivated and expresses that they would consider self-pay options<br>☐ Irrespective, patients should always be given the option to choose whether they wish to self-pay<br>☐ I do not prescribe provincially unfunded treatments<br>☐ Other (please describe below)
Please describe:	__________________________________
What proportion of your patients is estimated to currently be on a provincially unfunded anti-cancer systemic therapy? (This includes self-pay/private insurance and therapies obtained through compassionate access programs)	◯ Less than 10%<br>◯ 10-20%<br>◯ 20-40%<br>◯ More than 40%
Does the institution where you currently practice permit the administration of unfunded treatments/infusions in the center?	◯ Yes<br>◯ Yes -- but only for therapies funded by a manufacturer compassionate access program<br>◯ No -- unfunded therapies cannot be administered within my centre/hospital

Do you have access to a dedicated drug access navigator at your center?	◯ Yes<br>◯ No
Based on your understanding of the approval process and anticipated pricing reforms in Canada, what is your level of concern regarding the future likelihood of new drug funding over the next 5 years?	◯ EXTREMELY concerned - it will be increasingly difficult for new cancer therapies to be approved for public funding<br>◯ MODERATELY concerned<br>◯ SLIGHTLY concerned<br>◯ NOT AT ALL concerned - funding for new cancer therapies will improve in the future

Case Scenarios

Case #1

54-year-old female with MMR deficient (dMMR), pre-treated, metastatic endometrial cancer. ECOG performance status of 1.<br><br>Pembrolizumab monotherapy is Health Canada approved for the treatment of adults with endometrial cancer that has progressed following prior therapy and who have no satisfactory alternative treatment option, based on the KEYNOTE-158 study which demonstrated an ORR of 57% and 12-month OS rate of 73% for MSI-H/dMMR endometrial cancers.<br><br>It is not publicly funded and there is no manufacturer compassionate access program. Assume no clinical trials are available. The patient does not have private insurance.<br><br>At the list price, pembrolizumab is estimated to cost approximately $12,000 per month.<br><br>(Select all that apply)	☐ I would ALWAYS discuss the option of self-pay pembrolizumab (assuming it was an appropriate next line of treatment and patient has no contraindications)<br>☐ I WOULD discuss - if the patient expressed that they would consider self-pay options<br>☐ I WOULD discuss - if it could be administered within my own cancer center<br>☐ I WOULD discuss - if it could be administered at a private infusion clinic<br>☐ I would NOT discuss self-pay pembrolizumab with the patient as it is not funded<br>☐ Other (please describe in the comments sections below)
Please indicate your top 2 reasons for not offering treatment to the patient: (Select TWO answers)	☐ I am not convinced that this treatment offers a meaningful benefit<br>☐ I don't think the patient could afford it<br>☐ I am worried it would cause increased stress for the patient and their family<br>☐ Insufficient time to do all the necessary paperwork to order and organize the treatment<br>☐ Other (please describe in the comments section below)
Do you have any other comments regarding case #1?	______________________________

Case #2

54-year-old female with MMR proficient (pMMR), KRAS-mutated metastatic colorectal cancer (mCRC), ECOG performance status of 1. Disease progression after 11 months on first line FOLFIRI (5-flurocuracil, irinotecan) and bevacizumab and 6 months on second line FOLFOX (5-fluorouracin, oxaliplatin) chemotherapy.<br><br>Oral trifluridine/tipiracil is Health Canada approved for the treatment of adults with mCRC that has progressed on a fluoropyrimidine, oxaliplatin and irinotecan, based on phase III RECOURSE trial which demonstrated a median OS improvement of 2 months over best supportive care (HR 0.69, p=0.0001).<br><br>It is not publicly funded, and there is no manufacturer compassionate access program. Assume no clinical trials are available. The patient does not have private insurance.<br><br>At the list price, oral trifluridine/tipiracil is estimated to cost $5,000 per month.<br><br>(Select all that apply)	☐ I would ALWAYS discuss the option of self-pay trifluridine/tipieracil (assuming it was an appropriate next line of treatment and patient has no contraindications)<br>☐ I WOULD discuss - if the patient expressed that they would consider self-pay options<br>☐ I WOULD discuss - if it could be dispensed by my own cancer center pharmacy<br>☐ I would NOT discuss self-pay trifluridine/tipieracil with the patient as it is not funded<br>☐ Other (please describe in the comments section below)
Please indicate your top 2 reasons for not offering it to the patient: (Select TWO answers)	☐ I am not convinced that this treatment offers a meaningful benefit<br>☐ I don't think the patient could afford it<br>☐ I am worried it would cause increased stress for the patient and their family<br>☐ Insufficient time to do all the necessary paperwork to order and organize the treatment<br>☐ Other (please describe in the comments section below)
Do you have any other comments regarding case #2?	______________________________

Case #3

54-year-old female with NTRK fusion-positive, pre-treated, metastatic non-small cell lung cancer. ECOG performance status of 1.

Larotrectinib monotherapy is Health Canada approved for the treatment of adults with advanced solid tumours harbouring an NTRK gene fusion, based on a pooled analysis from three non-comparative phase I/II trials which demonstrated an ORR of 81%, and a median PFS of 28.3 months.

It is not publicly funded, and assume there is no manufacturer compassionate access program. Assume no clinical trials are available. The patient does not have private insurance.

At the list price, oral larotrectinib is estimated to cost approximately $20,000 per month.

(Select all that apply)

- ☐ I would ALWAYS discuss the option of self-pay larotrectinib (assuming it was an appropriate next line of treatment and patient has no contraindications)
- ☐ I WOULD discuss - if the patient expressed that they would consider self-pay options
- ☐ I WOULD discuss - if it could be dispensed by my own cancer center pharmacy
- ☐ I would NOT discuss self-pay larotrectinib with the patient as it is not funded
- ☐ Other (please describe in the comments section below)

Please indicate your top 2 reasons for not offering it to the patient: (Select TWO answers)

- ☐ I am not convinced that this treatment offers a meaningful benefit
- ☐ I don't think the patient could afford it
- ☐ I am worried it would cause increased stress for the patient and their family
- ☐ Insufficient time to do all the necessary paperwork to order and organize the treatment
- ☐ Other (please describe in the comments below)

Do you have any other comments regarding case #3?

____
